# Access authentication via blockchain in space information network

**DOI:** 10.1371/journal.pone.0291236

**Published:** 2024-03-07

**Authors:** Muhammad Arshad, Liu Jianwei, Muhammad Khalid, Waqar Khalid, Yue Cao, Fakhri Alam Khan

**Affiliations:** 1 School of Cyber Science and Technology, Beihang University, Beijing, China; 2 School of Computer Science, University of Hull, Hull, United Kingdom; 3 School of Cyber Science and Engineering, Wuhan University, Wuhan, China; 4 Department of Information and Computer Science, King Fahd University of Petroleum and Minerals, Dhahran, Saudi Arabia; 5 Shenzhen Research Institute of Wuhan University, Wuhan, China; 6 Interdisciplinary Research Centre for Intelligent Secure Systems, King Fahd University of Petroleum Minerals, Dhahran, Saudi Arabia; Xiamen University Malaysia, MALAYSIA

## Abstract

Space Information Network (SIN) has significant benefits of providing communication anywhere at any time. This feature offers an innovative way for conventional wireless customers to access enhanced internet services by using SIN. However, SIN’s characteristics, such as naked links and maximum signal latency, make it difficult to design efficient security and routing protocols, etc. Similarly, existing SIN authentication techniques can’t satisfy all of the essentials for secure communication, such as privacy leaks or rising authentication latency. The article aims to develop a novel blockchain-based access authentication mechanism for SIN. The proposed scheme uses a blockchain application, which has offered anonymity to mobile users while considering the satellites’ limited processing capacity. The proposed scheme uses a blockchain application, which offers anonymity to mobile users while considering the satellites’ limited processing capacity. The SIN gains the likelihood of far greater computational capacity devices as technology evolves. Since authenticating in SIN, the technique comprises three entities: low Earth orbit, mobile user, and network control centre. The proposed mutual authentication mechanism avoids the requirement of a ground station, resulting in less latency and overhead during mobile user authentication. Finally, the new blockchain-based authentication approach is being evaluated with AVISPA, a formal security tool. The simulation and performance study results illustrate that the proposed technique delivers efficient security characteristics such as low authentication latency, minimal signal overhead and less computational cost with group authentication.

## 1 Introduction

**Fig 1:**
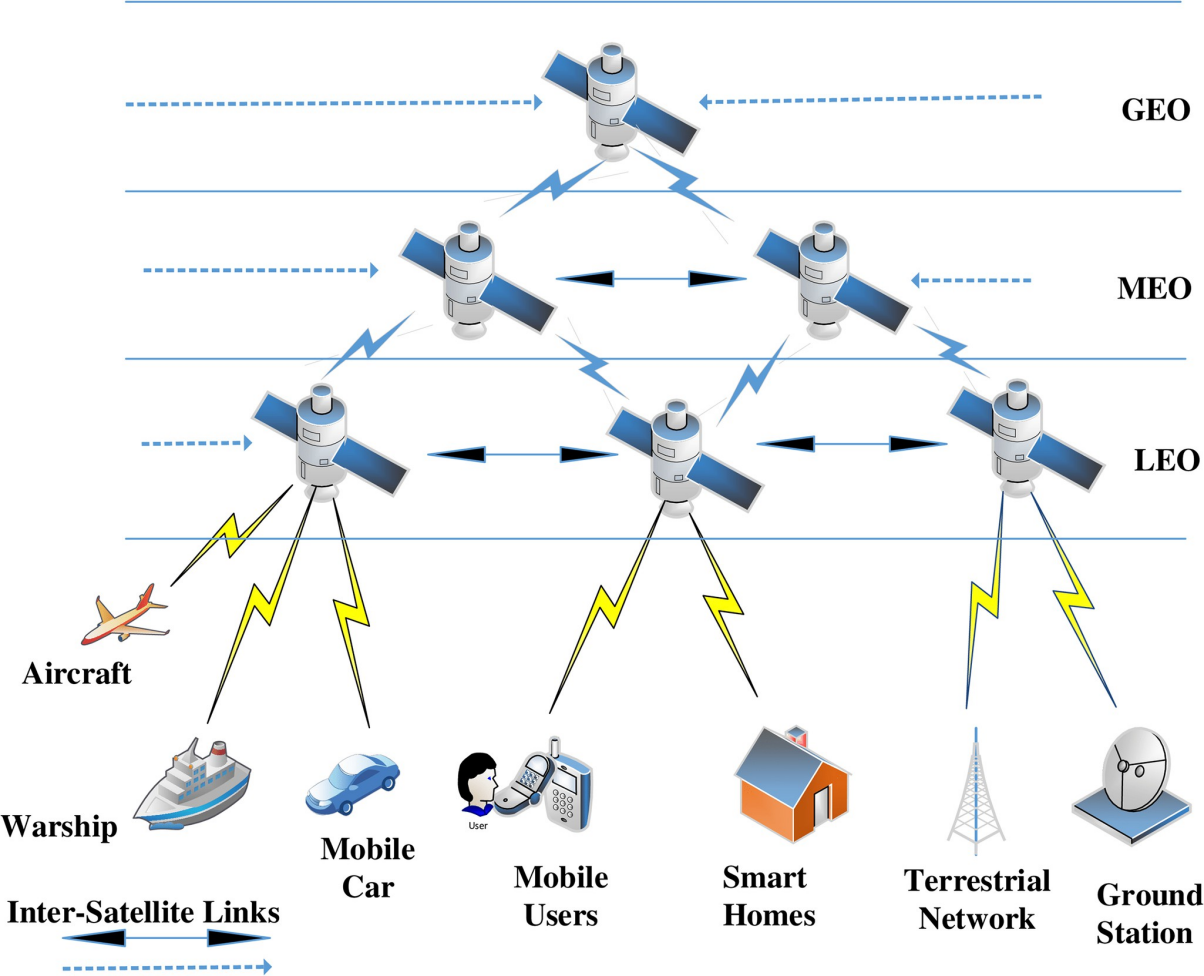
Architecture of SIN.

The open sharing of information for the globalization, urging communication anytime and anywhere is becoming more vital [1]. Space Information Network (SIN) has been practically implemented in real life, as satellites functioning as a relay to transmit electromagnetic waves accomplishes a broad range of transmissions. After this, SIN can be evolved for the Internet that connects satellites with terrestrial Internet carrying future space observation and global Internet access information [2]. In the last decade, SIN has gained enormous attention from researchers with the advancement of wireless communication technology. Generally, SIN consists of a satellite constellation system [3]. There are four levels of satellites based on their altitude: Medium Earth Orbit (MEO) satellites, Low Earth Orbit (LEO) satellites, Geosynchronous Earth Orbit (GEO) satellites, and ground-based stations. The GEO satellites are used for space backbone networks and are responsible for transmitting data between satellites and users. MEO satellites are typically responsible for monitoring the environment and earth assessment. The LEO satellites gather earth observation data and transmit to the earth base station as shown in Fig 1.

The ground networks (Terrestrial Networks) include surface nodes like Mobile cars, mobile terminals, smart homes, Aircraft, and warships [4]. SIN has various characteristics compared to Terrestrial networks, such as excessive link bit error rate, broad coverage, long latency, diversified network structure, and dynamic unreliable topology. The above-mentioned features generate additional security concerns for SIN. To counteract any malicious assault, the SIN should be protected with advanced protocols. Impersonation, the man in the middle, eavesdropping, replay assaults, and denial of service (DOS) attacks are examples of such attacks [5–7]. The most significant security features are confidentiality, user privacy, mutual access authentication, perfect backward/forward secrecy, and minimal computing cost [8, 9].

For quality of service (QoS) and security, a secure access authentication protocol is required for SIN. Conventional access authentication protocols are classified into two types: The centralized access authentication protocol and the mutual access authentication protocol. Centralized access authentication algorithms and verifying mobile users (MUs) at a trusted server required more exchange of messages. Nonetheless, the SIN system has a higher propagation delay between the ground station and the satellites. Fortunately, LEO satellites are relatively near the ground and have a less delay in the propagation of approximately 10 to 40 milliseconds in contrast to MEO and GEO satellites [10–12] since centralised protocols of authentication are incompatible with SIN. The mutual authentication protocol does not require the use of a third-party server for verification and MUs typically involve fewer message exchanges and lead to lower authentication delay [13–16]. Moreover, existing mutual access authentication mechanisms, on the other hand, are unsuitable for SIN as still they require time-consuming procedures (interactions) [17, 18]. In addition, the limited storage capacity and computation power of the corresponding nodes in SIN makes it hard to deploy such algorithms with high rate complexity. Therefore the new protocol should be lower complexity and efficient cryptographic strategy [19, 20].

2008 Satoshi Nakamoto proposed a decentralized cash system called Bitcoin. The system provides peer-to-peer electronic payment, producing secure and direct transactions between the communicating entities without deploying a trusted party. The procedure of bitcoin is based on a consensus algorithm to sustain a decentralized ledger known as a blockchain [21]. The data in ledgers are preserved from tempering, revision, and deletion. Blockchain is an open, decentralized system that records transactions between two users efficiently in a permanent way. Even though the blockchain system was designed for Bitcoin, however, we can use the blockchain application as transnational data sets duplicated and scattered over various nodes which are communicating in peer to peer network. The blockchain comprises a chain of blocks, and each block has a header and body. The body includes facts stored in plain text or cipher-text, and facts can be anything, such as financial transactions, health data, power plant data, images, etc. The header maintains information regarding the block, like Merkle Rootcite [22], which includes a hash of the block transactions, timestamp, transaction number, and the preceding block’s hash. Blockchains can be private such as Hyperledger Fabric [23], or public such as Bitcoin and Ethereum [24]. Ethereum represents a framework for decentralized applications as well as procedure programs known as smart contracts. Smart contracts are executed by participating nodes using the Ethereum Virtual Machine [24, 25]. The blockchain employs a protocol called proof of work (PoW) to resist modification attacks and enhance the Ethereum and Bitcoin security systems. The proof of work data procedure is expensive and time-consuming, but it makes it difficult for attackers who wish to falsify the previous block against an honest node.

As a lightweight and feasible solution to SIN, Ethereum must be used as a private blockchain. The participating nodes in a private blockchain work as a group, and the consensus algorithm, such as the proof of Work (PoW) scheme, is not needed for verification. The contributors to the consensus are subject to limitations on the private blockchain. The only nodes with the authority to confirm transactions are those chosen as trustful. Since reaching a consensus does not require a lot of computation, a private blockchain is neither time nor energy-consuming. The decentralization of blockchain is a principle adopted by the Practical Byzantine Fault Tolerance (PBFT) algorithm, which also ensures an improvement in fault tolerance and scalability. It reduces the communication complexity of the network by using a consistent hash algorithm to the group of nodes to reach consensus and prevent frequent communication between nodes [24, 26].

The resilience of private blockchain applications could also assist SIN [22]. In a real sense, since satellite instrument technology advances, it will be ready to execute more computations and permit for authentication inter-linkage with MUs. As a result, it is essential to use satellites instead of ground stations for access authentication control and limiting network access latency. These studies exerted a strong influence on the three major contributions raised in the article.

- We design a new blockchain-based mutual authentication protocol that allows for efficient mutual authentication for SAP and MUs. Compared to other authentication techniques, this approach offers the lowest signal overhead and authentication delay.
- We identify the important security requirements for the authentication protocol and build the mechanism that deploys blockchain applications on the SAP as well as on MUs for SIN to ensure secure communication during access authentication.
- Eventually, the AVISPA tool is used to affirm the blockchain-based authentication protocol. The security analysis and verification indicate that the blockchain-based access authentication protocol is successful regarding security requirements. The verification results further revealed that, when compared to existing authentication protocols, the proposed protocol provides a short running time, as well as a low computation cost for group and individual authentication.

The following sections are used to organize the remaining article. In section 2, we provide examples of the relevant studies and illustrate their performance and vulnerabilities. The mathematical requirements are introduced in Section 3, and the system architecture and security requirements are mentioned in Section 4. A blockchain-based access authentication algorithm was also comprehensive in section 5. The security comparisons and analysis are included in section 6. Section 7 explains the formal security verification using the AVISPA tool. In 8 and 9, respectively, performance comparison and conclusion are presented.

## 2 Related work

In this segment, we discuss an overall view of access authentication research both in traditional wireless networks and SIN.

### 2.1 Authentication protocol for conventional network

Public Key Infrastructure (PKI) is used in conventional networks to address authentication, but it is complicated and time-consuming in wireless networks, causing a long propagation delay and overhead for SIN. Besides that, the identity-based authentication protocol recommended in [27–29] required different identities, which is challenging for MUs since phones and tablets possess the limited capacity. Identity-based authentication mechanisms also rely on trusted authority, which may have an increased congestion single point of failure. For GSM authentication, other protocols, such as certificate-less public key cryptography (CL-PKC), are implied. The protocol generated both user-side and key generation centre keys. However, SAP may hurt user privacy. so all these schemes cannot be used for SIN. In conventional wireless networks, nevertheless, certain authentication algorithms take the user issue into consideration. For instance, [30] To give identification, a simple mobile authentication technique was provided, and group signatures have been employed. In [31–33] proposed a scheme to safeguard the privacy of MUs on networks with unreliable security. Nonetheless, these approaches are ineffective for SIN since there is a substantial signal propagation delay among ground stations and satellites. In [22] suggested an Internet of Things (IoT) access authentication mechanism based on blockchain for devices with storage, energy, and consumption restrictions.

### 2.2 Authentication protocols for SIN

In previous decades, researchers concentrated on access authentication in SIN. Cruickshank presented the first authentication scheme for SIN. The authentication technique uses a public key and a private key system to meet infrastructure requirements. Unfortunately, due to key size and more complex operations, this technique lacks the capability of fully protecting MUs confidentiality [34]. The bit-wise exclusive-r operation, a hash function, and string concatenation are the basis of the lightweight access authentication scheme that has been developed [3]. In [35] the unique logarithm problem and one-way hash function are used to create a new algorithm that defends against well-known malicious attacks, optimizes the two current techniques, and enhances security. In the interest of safeguarding against replay attacks, a nonce technique is also deployed. The [36, 37] distinguish security as imperative and present a new scheme that strengthens security while guarding user privacy. Although these protocols meet all of the SIN security requirements, but we are still unable to implement them since they have higher latency for signal propagation and may result in compromised user privacy and long access authentication delays.

In [17] develop an unnamed and quick roaming authentication protocol that utilizes group signatures to protect MUs privacy. For SIN, the scheme proposed proxy signature access-based authentication. To reduce long authentication delays, authentication is incorporated between MUs and satellite nodes [38]. A simple scheme for access authentication based on an automatic procedure for updating users’ short-term identities. The protocol has been separated into two phases: registration and authentication. Nevertheless, due to the complicated operation, this scheme generates more overhead and latency [39]. In [40], establish an access authentication algorithm that is both efficient and secure, perfect with a handover scheme. Satellites can verify MUs while avoiding NCC, but they can’t reduce latency and congestion by running additional authentication iterations. In [41] proposed a new protocol for SIN that uses encryption-based key update and mutual authentication. The scheme performance is evaluated using the reply and man-in-the-middle attacks. Despite this, all current SIN access authentication algorithms assume that the satellite’s role is merely a pass-through between MUs and ground stations and that it is only in charge of forwarding data. As a consequence, access authentication is employed between MUs and ground equipment, resulting in increased authentication latency. Furthermore, privacy information leakage is a serious issue for the SIN authentication system, whereas MUs privacy safeguarding is complicated. As a result, these protocols are time-consuming and unsuitable for MUs of limited computation speed.

The security standards are met by existing authentication protocols based on decentralised or mutual authentication techniques. Furthermore, because fewer communications are sent, these techniques have a shorter latency than centralized authentication protocols. However, these schemes employ complex operations that further result in enhanced propagation delay, high computational, and overhead. Consequently, we will design a lightweight blockchain-based authentication protocol that exchanges too few messages while preserving SIN security.

## 3 Mathematical preliminaries

The following sections present the mathematical prerequisites for the proposed blockchain-based authentication algorithm. We began by illustrating Elliptic Curve Cryptography (ECC), Elliptic Curve Key Pair Generation, and the Elliptic Curve Digital Signature (ECDS) algorithm.

### 3.1 Elliptic curve cryptography

Elliptic Curve Cryptography (ECC) is a security algorithm used to protect blockchains. It is a lightweight security protocol and an asymmetric public key cryptography algorithm. The ECC generates public and private keys for secure data communication, as well as digital signature services for message signing. When compared to other security algorithms, the ECC algorithm consumed less power on the device [42]. The key advantage of ECC over the other public key cryptography since it has a smaller key size and offers the same level of security 256-bit key as such RSA (Rivest, Shamir, Adelman) 3,072-bit key [43].

#### 3.1.1 Key pair generation

For the two devices E and F key generation.

Let’s assume that users E and F private keys are SE and SF, respectively. Random numbers less than n, where n is a domain variable, make up the private keys.

Let presume *QE* = *SE* * *G* and *QF* = *SF* * *G* is the public key of device E and F respectively, where G is a domain variable.

E and F mutually exchanged their public keys

E calculate *K* = (*iK*, *jK*) = SE*QF

F calculate *L* = (*iL*, *jL*) = SF*QE

Since *K* = *L*, shared confidential is known as *iK*

#### 3.1.2 Elliptic curve digital signature algorithm

The public key algorithm used for a digital signature is called the digital signature standard (DSA). The parameters for the elliptic curve the domain must be agreed upon by the two devices before a signed message (M) can be transmitted from device E to devise F. The ECDSA algorithm’s additional information are illustrated here.

Assume device E signs M and delivers it to device F.

Let SE be E’ private key.

Determine *M* = *Hash*(*M*), where the hash is a hash function, like SHA-1

choose a random integer k like 0 < *k* < *n*

Determine *r* = *x*_1_ mod n, where (*x*_1_, *y*_1_) = k*G

Determine *s* = *k* − 1(*M* + SE*r)*modn*

The signature is the pair of (*r*, *s*)

## 4 System and security model

### 4.1 System model

**Fig 2:**
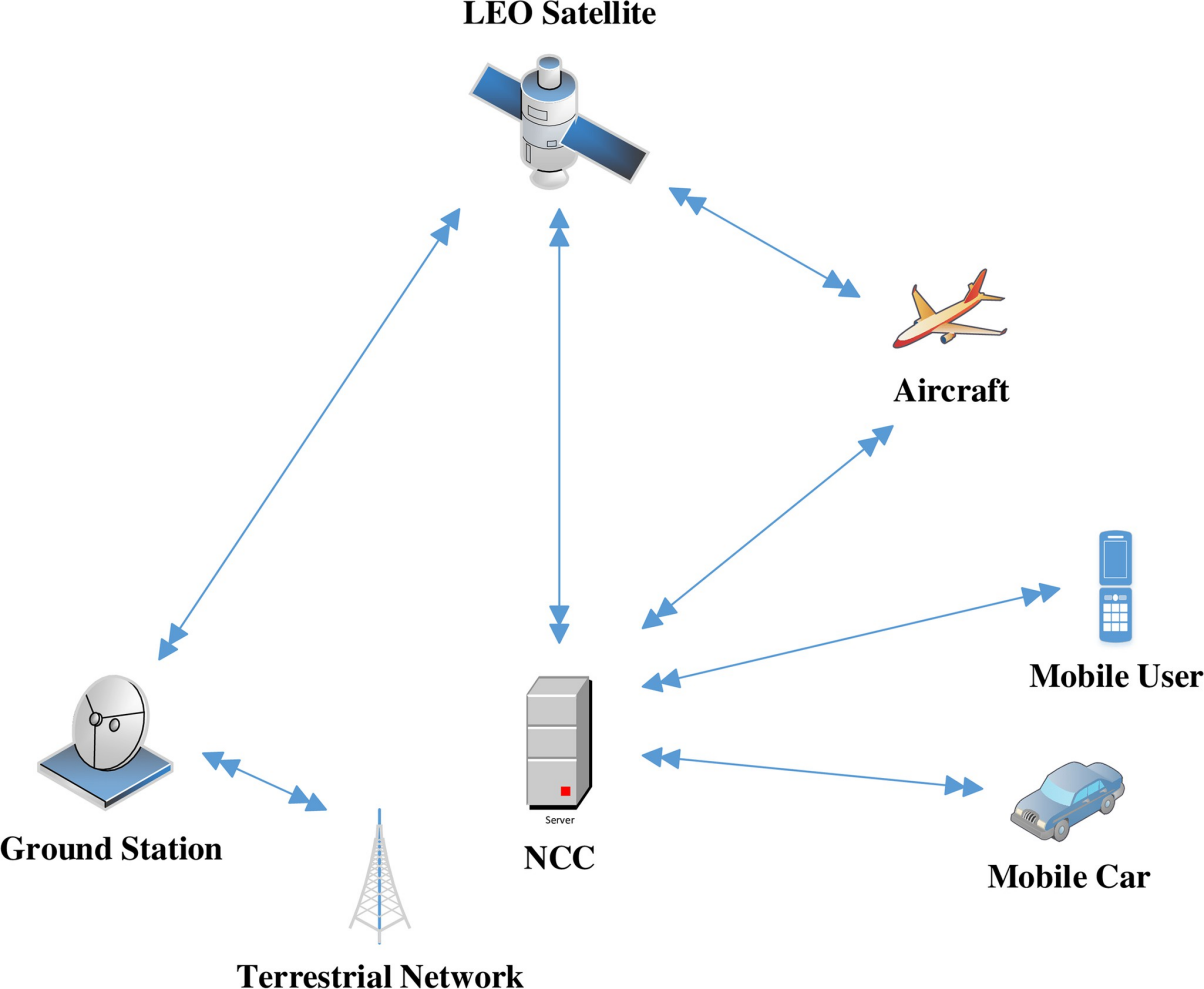
System model of our proposed protocol.

The access authentication protocol comprises the following communication entities MUs, ground station/gateway, Low Earth Orbit (LEO), also known as SAP, and Network Control Centre (NCC). In the conventional strategy, MUs are first registered with the NCC. The MUs could be anything, including a car, ship, aeroplane, or mobile phone. LEO typically controls message forwarding between MUs and the ground station and being close to the earth. Nevertheless, during access authentication, such as in [17, 40] these sorts of communication induce additional propagation delay and signal overhead. In the blockchain authentication system, we consequently assume that NCC, MU, and LEO satellites are interacting directly with no assistance from a ground station, as shown in Fig 2. As a consequence, compared to other access authentication techniques, it causes less propagation latency and signals overhead. The proposed protocol uses a private, immutable blockchain that is lightweight, secure, and used for authentication between MUs and SAP. It leverages the PBFT consensus technique for the decentralization of fault-tolerant and scalable blockchain networks. In order to come to an understanding between the MUs and SAP, it adopts a consistent hashing technique, which decreases the communication complexity of the network. This blockchain [26] uses the PBFT vote-counting technique to ensure that the group’s consensus is fault-tolerant.

**Fig 3:**
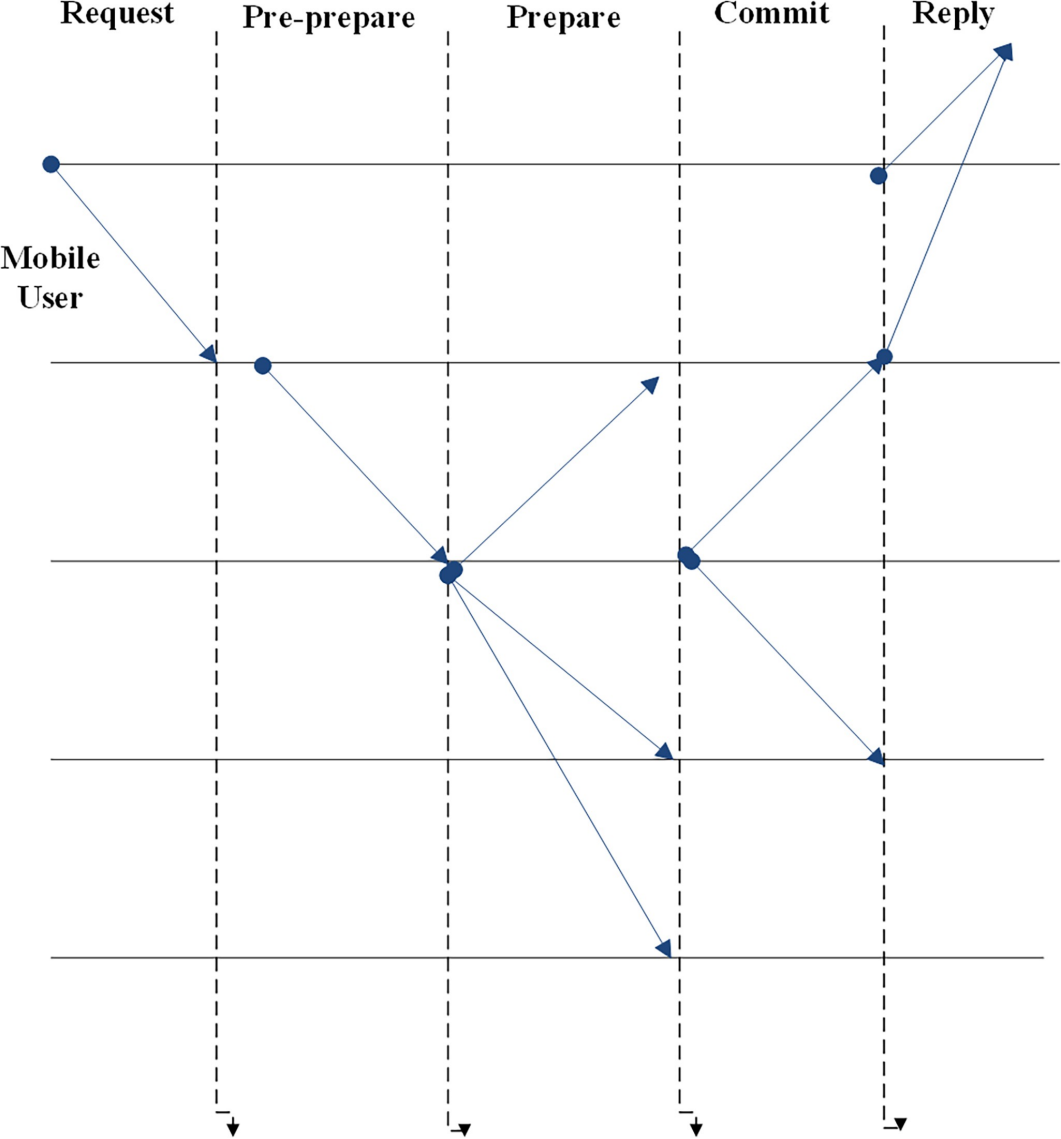
PBFT consensus protocol.

The steps in the PBFT consensus algorithm are as follows:

MUs send an alert to SAP. The request is subsequently sent to the blockchain nodes by SAP. The requests issued by the MUs get processed by all blockchain nodes, who then respond to the MUs. The MUs await for (f + 1) identical responses from each node. Where f is the number of potential malicious nodes. The PBFT algorithm is divided into three phases:

- Pre-prepare phase: Blockchain nodes acquire a message that the SAP node already generated.
- Prepare: The blockchain nodes send the prepared message as a reaction to MUs and SAP after receiving the pre-prepared message from SAP. Only once an SAP has seen the (2f + 1) number of prepared messages from other nodes and received pre-prepared messages from the blockchain is it considered prepared.
- Commit: If both the MUs and SAP are willing, they would then send a commit message. When MUs receive (f + 1) commit messages, they begin communicating. The step of the PBFT consensus algorithm has been illustrated in Fig 3.

### 4.2 Security model

The proposed approach includes the following security constraints. While deploying the blockchain system, the proposed protocol presupposed a reliable line of communication between LEO and NCC. It is extremely difficult for adversaries to compromise each of the devices. The technique also assumes that the MUs, NCC, and LEO have encrypted channels for communication. Since the NCC, LEO, and MUs all communicate with one another, we supposed that multiple adversaries could disrupt or alter the communication signals while MUs are accessing the SIN, or the adversaries are trying to compromise the proposed blockchain-based authentication protocol.

#### 4.2.1 Security prerequisites

The following requirements must be fulfilled by the blockchain-based authentication protocol for SIN.

**Mutual Authentication:** The proposed access authentication system should be able to authenticate one another, recognize unauthorized MUs, and deny their requests, according to the first requirement.

**Anonymity:** No one can reveal the real identity from the access authentication communication apart from NCC. The user’s location privacy could be protected in the scheme to maintain anonymity.

**UnLikability:** The adversaries are incapable to combine various authentications with the goal to acquire information for the same MUs.

**Key Agreement:** A key agreement between LEO and MUs ought to be feasible with the blockchain-based authentication protocol.

**Backward/Forward Secrecy:** It is essential that the security of the upcoming session key can’t be impacted by the disclosure of current key information [17].

## 5 Proposed access authentication scheme via blockchain

Instead of using the conventional approach for SIN, the proposed blockchain-based access authentication protocol is a decentralized and more secure. According to Fig 2, the proposed communication model consists of three entities MUs, LEO, which will function as a satellite access point (SAP) and NCC. The Table 1 depicts the various notations used in the proposed protocol.

**Table 1:** Notations used in our protocol.

Notation	Definition
Muk	MUs public key
Sak	SAP public key
Nck	NCC public key
Bck	Blockchain public key
Nonce	Random Number
H	Hash Function
G	Domain Variable
*P*_*mu*_	Proof of MUs
*P*_*sap*_	Proof of SAP
*Lt*_*k*_	Lifetime of key
id	MU Identity
N	Number of Mobile Users
Witness	Uses for strong authentication

**Fig 4:**
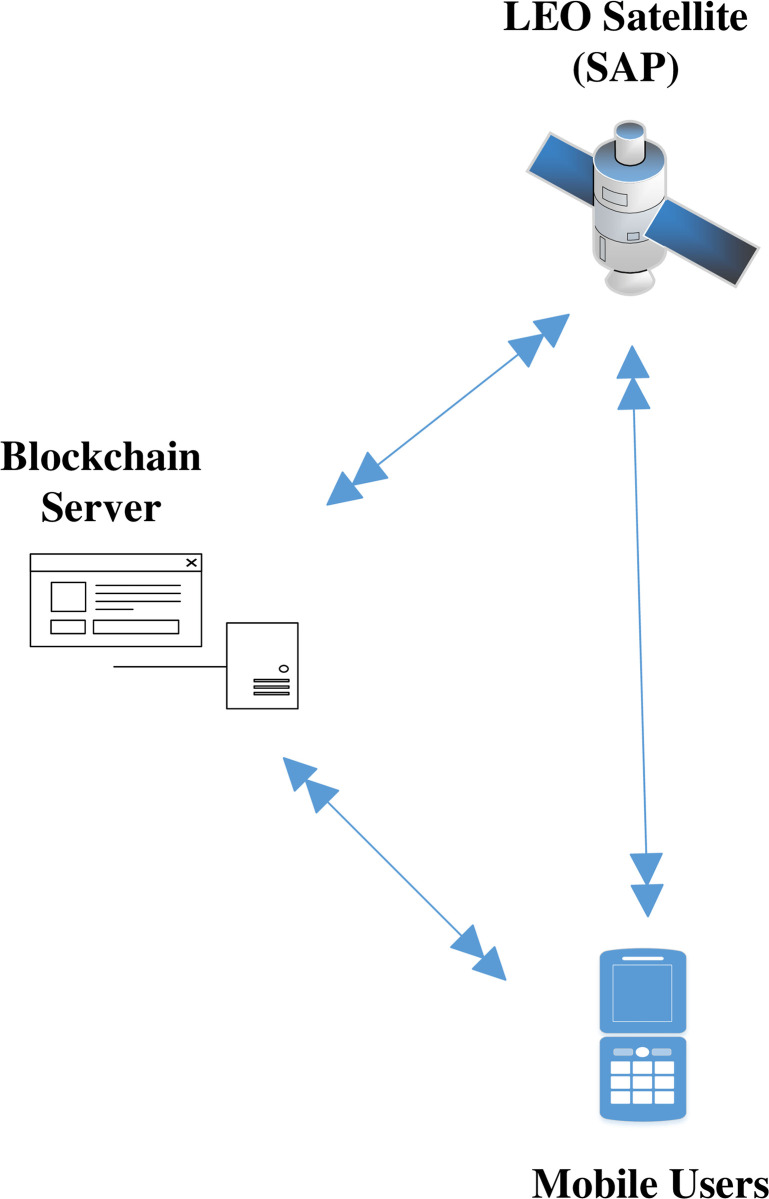
Blockchain based mutual authentication for MUs.

Our strategy consisted of two parts. The initial stages of MUs and LEO satellite registration with NCC have been briefly described. The second phase involves a comprehensive demonstration of a SIN blockchain-based mutual authentication system as demonstrated in Fig 4.

### 5.1 Initial phase

In the initial phase, Eq (1) shows how MUs generate the private key (*MU*.*private* − *key*) and the corresponding public key (*MU*.*Public* − *key*) during the initial phase. As shown in Eq (2), the NCC also creates his private key, (*NCC*.*Private* − *key*), and the corresponding public key, (*NCC*.*Public* − *key*). As shown in the Eq (3), SAP also created his private key (*SAP*.*Private* − *key*) and corresponding public key (*SAP*.*Public* − *key*). G is a domain parameter that was talked about in (section III)

$$\begin{array}{c} MU.Muk=MU.inv(Muk)*G \end{array}$$ (1)

$$\begin{array}{c} NCC.Nck=NCC.inv(Nck)*G \end{array}$$ (2)

$$\begin{array}{c} SAP.Sak=SAP.inv(Sak)*G \end{array}$$ (3)

#### 5.1.1 Mobile user registration

**Fig 5:**
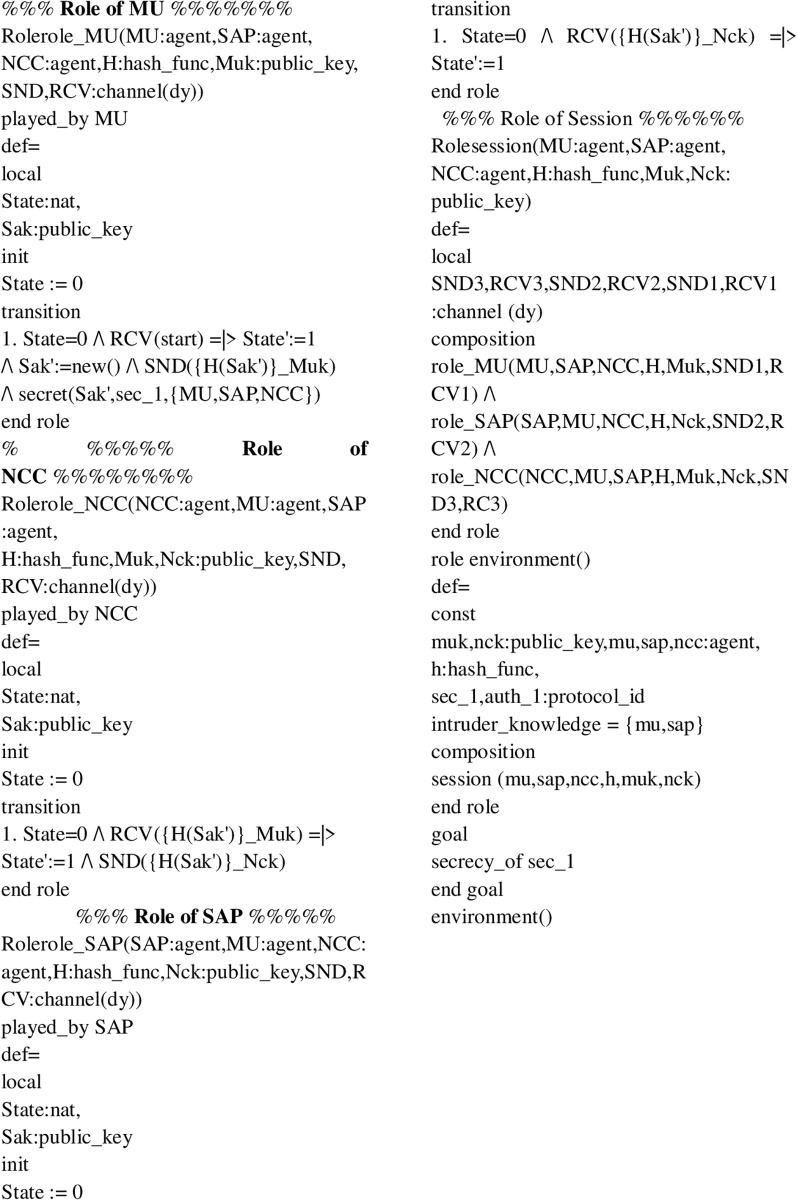
MUs and SAP registration to NCC.

In the initial stage, MUs use a secure channel to communicate a registration request to NCC along with a public key. The MUs compute the values listed as (*P*_*MU*_‖*Lt*_*K*_‖*H*‖(*SAP*_*ID*_)‖*MU*_*ID*_) and send them to the NCC. Following receipt from the MUs, the NCC verifies the MU public key, the life time of the (*Lt*_*k*_) key, and the (*P*_*MU*_) proof, where *MU*_*ID*_ includes both the MUs’ public and private keys. The confidentiality of MU’s keys is guaranteed by a secrecy parameter, as shown in Table 2 and Fig 5. Additionally, NCC validates his credentials and sends the subsequent(*P*_*MU*_‖*Lt*_*K*_‖*H*‖*SAP*_*ID*_‖*NCC*_*ID*_) parameters to SAP in order to start blockchain-based mutual authentication.

**Table 2:** MUs and SAP satellite registration to network control center.

MU	NCC	SAP
*Select*, *MUs*, *SAP*, *NCC*, *Hash*−*Func* (*P*_*MU*_‖*Lt*_*K*_‖*H*‖(*NCC*_*ID*_)‖*MU*_*ID*_) *Secret* = *NCC*_*ID*_, *Secrecy*(*MU*, *SAP*, *NCC*)		
	*Select*, *NCC*, *MUs*, *SAP*, *Hash*−*Func*. *Compute* = (*P*_*MU*_‖*Lt*_*K*_‖*H*‖(*NCC*_*ID*_)‖*MU*_*ID*_) *Compute* = (*P*_*SAP*_‖*Lt*_*K*_‖*H*‖*NCC*_*ID*_‖*SAP*_*ID*_)	
		*Select*, *SAP*, *NCC*, *MUs*, *Hash−Func*. *Compute* = (*P*_*MU*_‖*Lt*_*K*_‖*H*‖(*NCC*_*ID*_)‖*SAP*_*ID*_) *Secret* = *NCC*_*ID*_, *Secrecy*(*SAP*, *SAP*, *NCC*)

#### 5.1.2 Satellite registration

Each SAP also require an NCC to verify its identification. Since, SAP sends a registration request to NCC in the amount of (*P*_*SAP*_‖*Lt*_*k*_‖*H*‖(*NCC*_*ID*_)‖*SAP*_*ID*_). Where (*P*_*SAP*_ is a Proof for SAP and *Lt*_*k*_ is the lifetime of SAP including a public key and private key for SAP. Where *Lt*_*SAP*_ is a lifetime of *SAP*_*ID*_) SAP key. The confidentiality of the SAP keys, as shown in Table 2, and Fig 5, is guaranteed by a secrecy parameter. Additionally, SAP sends NCC his credentials parameters, which include the following:(*P*_*SAP*_‖*Lt*_*K*_‖*H*‖*NCC*_*ID*_‖*SAP*_*ID*_). Finally, NCC validated each of these factors in order to launch a secure authentication procedure between SAP and MUs.

### 5.2 Blockchain-based mutual authentication

A MUs sends a request to SAP, which triggers the blockchain authentication process Table 3 provides a summary of the mutual authentication process based on blockchain.

**Table 3:** Blockchain-based mutual authentication protocol for SIN.

MU	Blockchain	SAP
*Select*, *MUs*, *SAP*, *Pubkey*,*Specialidl*, *MUID*, *id*, *Comp*,*HashFunc*, *Authen*_*r*_*equest*. (*id* = *Nonce*‖*Authenrequest* = *Nonce* *Secrecy* = (*id*‖*sec*_1_‖*MU*, *SAP*)*Witness*(*MU*, *SAP*‖*Auth*_1_‖*id*‖*Authen* − *request* *Compute* = *SND*(*Authen* − *request* ‖*inv*(*Muk*)‖(*Sak*)		
		*Select*, *SAP*, *NCC*, *MUs*, *Blockchain*, *Pubkey*, *MUID*, *id*, *Comp*, *Authen* − *request*, *HashFunc*, *Compute* = *RCVAuthen* − *request* ‖*inv*(*Muk*)‖*Sak* *id* = *Nonce* *Compute* = *SND*(*H*‖*id*‖*inv*(*Sak*)‖*Bck*)
	*SelectBlockchain*, *SAP*, *NCC*, *MUs*, *HashFunc*, *Pubkey*, *MUID*, *id*, *Comp*, *Authen* − *request* *Compute* = *RCV*(*HashFunc*‖*id*‖*inv*(*Sak*)‖*Bck*) *Compute* = *SNDH*‖(*id*‖*MUID*‖*Muk*) *Compute* = *SNDH*‖(*id*‖*MUID*‖*Sak*)	
*Compute* = *RCVH*‖(*id*‖*MUID*‖*Muk*)		
		*Compute* = *RCVH*‖(*id*‖*MUID*‖*Sak*)
*Compute* = *SND*(*SpecialidI*‖*Sak*)		
		*Compute* = *RCV*(*SpecialidI*‖*Sak*)*Comp* = *Nonce* *secrecy*(*Comp*‖*sec* − 1‖*MUs*, *SAP*)*witness*(*MUs*, *SAP*‖*auth* − 1‖*Comp*) *Compute* = *SND*(*Comp*‖*Muk*)
*Compute* = *RCV*(*Comp*‖*Muk*)		

Mutual authentication protocol based on blockchains declares identities as variables. These variables include *id*, *Authen* − *request*, *Comp*, *MUID* and *H* which performs a hash function. While the *authen* − *request* acts as a parameter for a mutual authentication request to SAP, the *specialid* serves as an identifier for MUs. The *MUID* is another identification parameter that works with a blockchain-based authentication. For the successful completion of the blockchain-based mutual authentication system, the last *comp* parameter function has been used. The blockchain also declares various identities and variables, along with *Bck* as a public key. The SAP declares *Sak* as a public key, identities, and variables in a manner similar to MUs and blockchain, which have all been covered previously.

#### 5.2.1 Authentication request

To ensure confidentiality, in the first step, MUs generate random numbers for the variables *id* and (*Authen* − *request*). The random number generation process is illustrated by the (*id* = *Nonce*‖*Authen* − *request* = *Nonce*) in Table 3 while nonce is used for random numbers. The expression *Secrecy* = (*id*‖*sec* − 1‖*MU*, *SAP*) illustrates the confidentiality of data transmission between MUs and SAP. The protocol uses *Witness*(*MU*, *SAP*‖*Auth*1‖*id*‖*Authen* − *request*), whereas the term *Witness* indicates a strong authentication. A MU’s expression for an authentication request is (*Authen* − *request*‖*inv*(*Muk*)‖*Sak*) where *Sak* is the public key of SAP and *inv*(*Muk*) is the private key of MUs. These parameters are concatenated by the MUs and forwarded to SAP for authentication. The receiving message contains the parameters from the expression (*Authen* − *request*‖*inv*(*Muk*)‖*Sak*). Where the *inv*(*Muk*) MUs digital signature and the credential of MUs containing authentication requests have all been verified by the SAP.

#### 5.2.2 Blockchain authentication

The SAP uses *id* = *Nonce* to generate a random number for a special ID. Furthermore, SAP creates a transaction expression (*H*‖*id*‖*inv*(*Sak*)‖*Bck*) for the blockchain. where *inv*(*Sak*) is the private key for SAP, and (*Bck*) is the public key for the blockchain. This transaction goes via a secure channel to the blockchain.

The blockchain has accepted a transaction from SAP satellite with the expression (*H*‖*id*‖*inv*(*Sak*)‖*Bck*). Where *inv*(*Sak*) is a digital signature formed by SAP and *Bck* is the public key for the blockchain as demonstrated in the Table 3. A fundamental feature of blockchain applications is the hashing of SAP credentials. Afterward, following a successful check of the SAP satellite credentials, blockchain generated two transaction messages for MUs and SAP satellite. The first transaction for MUs is produced using the expression (*id*‖*MUID*‖*Muk*). Where *MUID* is a unique identifier created by blockchain specifically for MUs and *Muk* is their public key. A secure channel has been used to forward the blockchain transaction to MUs. Similar to the first transaction, the second expression (*id*‖*MUID*‖*Sak*) was produced by blockchain for the SAP. The parameters are *id*, which stands for a MU’s special id, and *Sak*, which remains for a SAP satellite public key. Additionally, a secure channel is used to transmit this transaction to SAP. Furthermore, MUs developed another special id referred (*SpecialidI*) to shield the identity from intruders. As a confirmation message, SAP acquires the special id concatenated with the public key of SAP (*Sak*) and (*SpecialidI*‖*Sak*).

In the final step, *Comp* = *Nonce* is employed to generate a random number for SAP satellite parameter function. Despite the fact that this information is communicated through a secure channel, a random integer is generated to avoid real identity guessing from intruders. In order to maintain confidentiality between MUs and SAP, the *secrecy*(*Comp*‖*sec* − 1‖*MU*, *SAP*) is in operation. SAP also has been using the *witness*(*MU*, *SAP*‖*auth* − 1‖*Comp*) parameter to provide robust mutual authentication between SAP satellite and MUs. For MUs, the SAP induces (*Comp*‖*Muk*), where the comp parameter proves the completion of the blockchain-based mutual authentication. SAP initiates sharing information with MUs after having completed blockchain-based authentication processes.

## 6 Comparison and security analysis

The resistance of the blockchain-based authentication scheme to different security attacks has been evaluated in this section.

### 6.1 Mutual authentication

The proposed blockchain application was used to achieve mutual authentication between MUs and SAP. SAP could authenticate MUs by referring over expression (*H*‖*id*‖*MUID*‖*Sak*). As a consequence of the blockchain’s strong resistance to security attacks, malicious nodes can’t gather information about the private key of the MUs from his/her public values. As a consequence, it is impossible to generate an accurate access request message without understanding the MUs’ private key. Only lawful MUs can generate the correct exact tuples expression *H*‖(*id*‖*MUID*‖*Muk*) to recognize mutual authentication. Similarly, MUs make sure legitimacy of the SAP by examining the expressions *H*‖(*id*‖*MUID*‖*Sak*) and (*Comp*‖*Muk*). Since solely a valid SAP with a valid private key can initiate accurate feedback. As a result, it has been demonstrated that proposed scheme can provide secure mutual access authentication between MUs and SAP.

### 6.2 Identity anonymity

The expression (*Authen* − *request*‖*inv*(*Muk*)‖*Sak*) illustrate in the proposed blockchain-based authentication scheme, where authentication request (*Authen* − *request*) acts as a random number and digital signature with the SAP satellite. Likewise, SAP has sent the MUs credential (*H*‖*id*‖*inv*(*Sak*)‖*Bck*) as a random number to protect identity anonymity. Furthermore, by utilizing a hash function, blockchain provides strong anonymity. Besides that, it is remarkably difficult for an attacker to disassemble the blockchain in order to generate MUs private key. To ensure MUs identity anonymity, a blockchain-based mutual authentication protocol utilises multiple identities in each transaction.

### 6.3 Unlinkability

The proposed blockchain authentication scheme has used a random number in registration as well as distinct identities during blockchain-based authentication such as *Authen* − *request*, *id* *MUID*, and *specialidl* authenticating MUs with SAP satellite. As a consequence, the proposed scheme prevents adversaries from learning about the same MUs. Since the proposed protocol guarantees MUs unlinkability at all times. Eventually, in the presence of trusted blockchain-based authentication outside adversaries can’t detect the actions of MUs.

### 6.4 Key backward/forward secrecy

To combat the problem of key forward and backward confidentiality between MUs and SAP. The authentication scheme incorporates different MUs identities and random numbers. According to methodology, (*id* = *Nonce*‖*Authenrequest* = *Nonce*) authorised requests and MUs identification (id), random variables are used, and these random values are concatenated with a MUs digital signature and SAP public key, according to in the expression (*Authen* − *request*)‖*inv*(*Muk*)‖(*Sak*) in Table 3. As a result, an attacker would have a less time guessing MUs private key. Similarly, SAP generates a random number *id* = *Nonceid* = *Nonce* once transmitting authentication (*H*‖*id*‖*inv*(*Sak*) ‖*Bck*) credentials to blockchain. As a consequence, our protocol ensures key forward and backward secrecy for MUs and SAP communication. Suppose a MU’s private key is compromised and messages are intercepted by an adversary. In the presence of a blockchain application, an attacker cannot easily a MUs private key from the public key. In other words, the adversaries’ knowledge is insufficient to compute previous MUs’ private keys.

### 6.5 Effectiveness against other attacks

The proposed blockchain-based authentication protocol is also resistant to the following known attacks.

#### 6.5.1 Impersonation attack

Assume adversary B intends to impersonate genuine A in order to compromise the blockchain authentication algorithm in this scheme. As the attacker must generate an authentication request that satisfied the equation *H*(*P*_*MU*_‖*Lt*_*k*_‖*H*‖(*NCC*_*id*_)‖*Muk*), where *P*_*MU*_ is the MUs proof, *Lt*_*k*_ is the MUs key lifetime, and *Muk* is the public key. The aforementioned equation is employed during the registration phase, according to Table 2. If the lifetime of the key and proof of MUs are produced by himself, and the data is generated via eavesdropping, B will be successful in thwarting the SIN, and he/she is the legitimate A. B, on the other hand, could not generate a genuine random number for the authentication request expression (*Authen* − *request*‖*inv*(*Muk*)‖ *Sak*) without knowledge of user A genuine private key. Furthermore, adversaries spoof valid authenticated MUs, breaching blockchain security unless a corresponding private key is acquired, which is a difficult task. It is impossible to generate a genuine private key in the presence of a hash function’s collision-resistant and one-way features. As a result, the proposed protocol is secure against masquerade attacks.

#### 6.5.2 Stolen verifier attack

The blockchain application is an encrypted verifier that is constantly running on the blockchain server, the proposed scheme is effective in resisting the stolen verifier attack.

#### 6.5.3 Replay attack

The proposed scheme resists replay attacks by introducing *PMU* proof for MUs and *LtK* lifetime of the MUs key. By implementing a timestamp value, the scheme is mitigate replay attacks. It has been indicated that registration phase data containing a timestamp value *LtK* is hashed to produce *H*(*P*_*MU*_‖*Lt*_*K*_‖*H*‖(*NCC*_*ID*_)‖*Muk*). For example, if an attacker obstructs and replays the authentication message, the SAP satellite can detect this sort of attack by examining the validity of the key’s lifetime. Since the collision-resistant and one-way characteristics of the hash function in blockchain, an attacker could not generate illegal request messages by modifying the lifetime of key value in a new authentication process. As a result, the algorithm safeguards against replay attacks.

## 7 Validation of orthodox security using the AVISPA tool: Verification evaluation

In this section, We will investigate the formal security verification of a blockchain-based mutual authentication protocol using an Ubuntu-based tool that automates the validation of internet security protocols and applications (AVISPA) [44, 45]. To perform formal security verification, the AVISPA tool employs a widely authorized security protocol animator (SPAN). AVISPA is a four-backend press-button mechanism. Specifically, “constrained logic-based attack searcher (CL-AtSe), on the fly model checker (OFMC), SAT-based model checker (SATMC), and tree auto-meta deploy on the automatic approximation for security protocol analysis” (TA4SP).

What circumstances make the test algorithm to an attack, or why is the test unverifiable? In the AVISPA tool, the high-level protocol specification language (HLPSL) is used to execute a security protocol intended to test whether the proposed algorithm is unsafe or safe while executing one of the four backends [46]. HLPSL is a “role-based language,” with two types of roles: composition and basic. The basic role denoted several algorithm participant entities. The composition roles depict the various scenarios found in the basic roles. In HLPSL, the attacker is specified using a security model known as the Dolev-Yao (DY) model. As a consequence, an attacker can play a legitimate role. An intruder is always participating in the verification as one of the basic lawful roles and is always visible through i. The algorithm’s HLPSL definitions are translated into the “intermediate format (IF)” by using the HLPSL2IF translator. The IF is then loaded into one of the four backends accessible in AVISPA to yield output format (OF). The OF has the following features [47].

SUMMARY: It demonstrates whether the simulated technique is unsafe, safe, or the outcome is unknown.

DETAILS: The details of “why the verification of an algorithm is over as safe, or in which circumstances the verified algorithm is vulnerable to an attack.

Algorithm: It locates the IF’s “HLPSL definitions of the intended algorithm.

GOAL: It depicts the goal of the results as achieved by the AVISPA tool utilizing HLPSL definitions.

BACKEND: It consists of the back-end names that are used for the result, which are TA4SP, OFMC, SATMC, and CL-AtSe.

The final part includes the detection of algorithm vulnerabilities, if any, along with useful comments and statistics [48].

**Fig 6:**
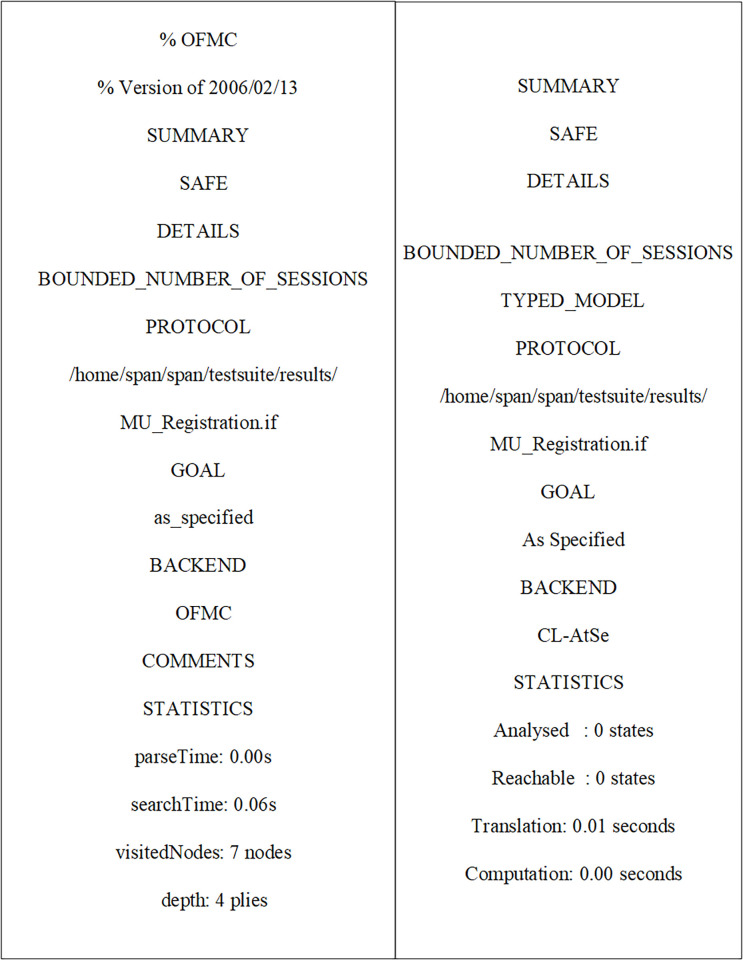
Verification results of the MUs and SAP registration to NCC using OFMC and CL-AtSe.

**Fig 7:**
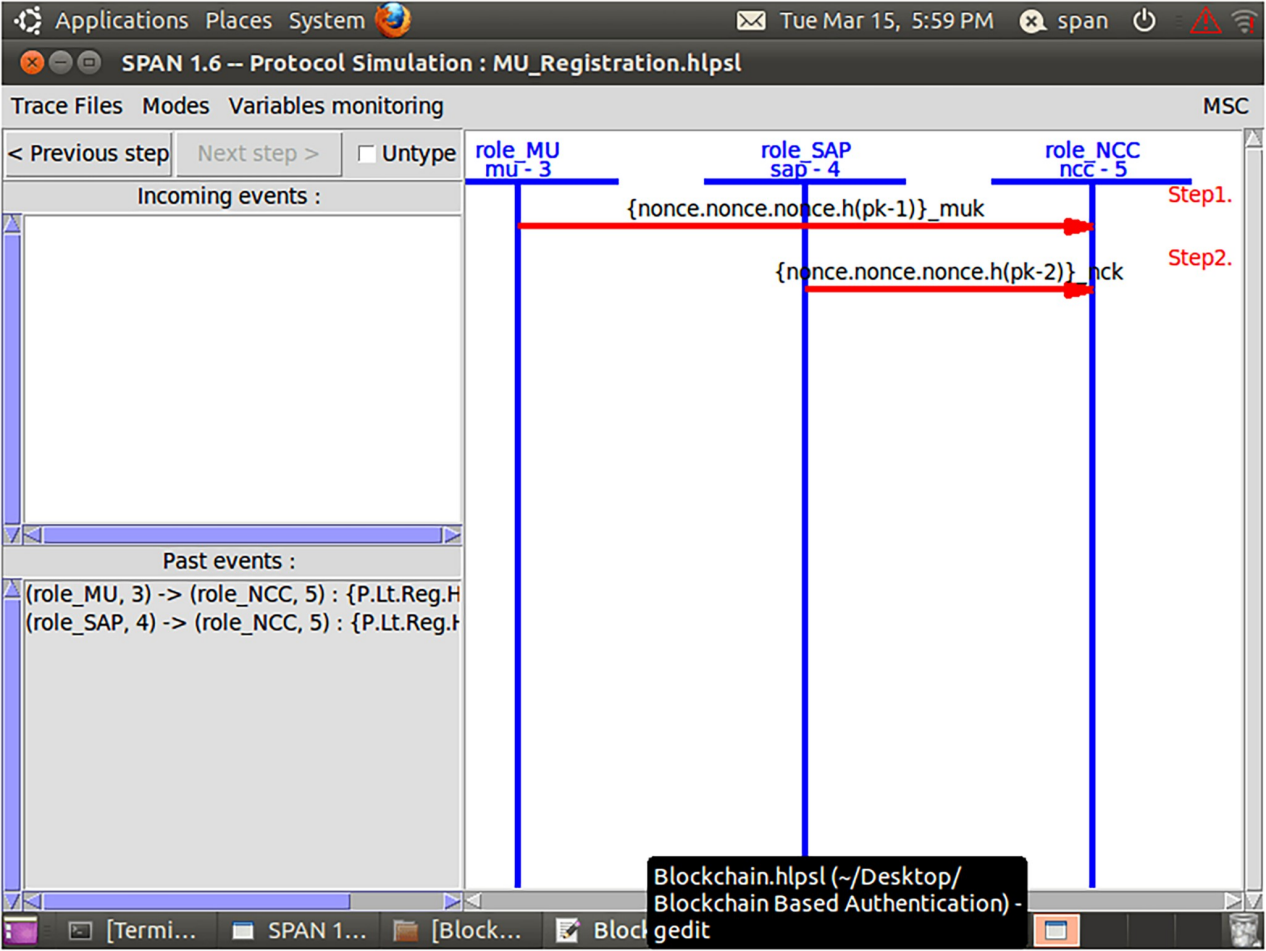
Verification for MUs and SAP registration using AVISPA tool.

**Fig 8:**
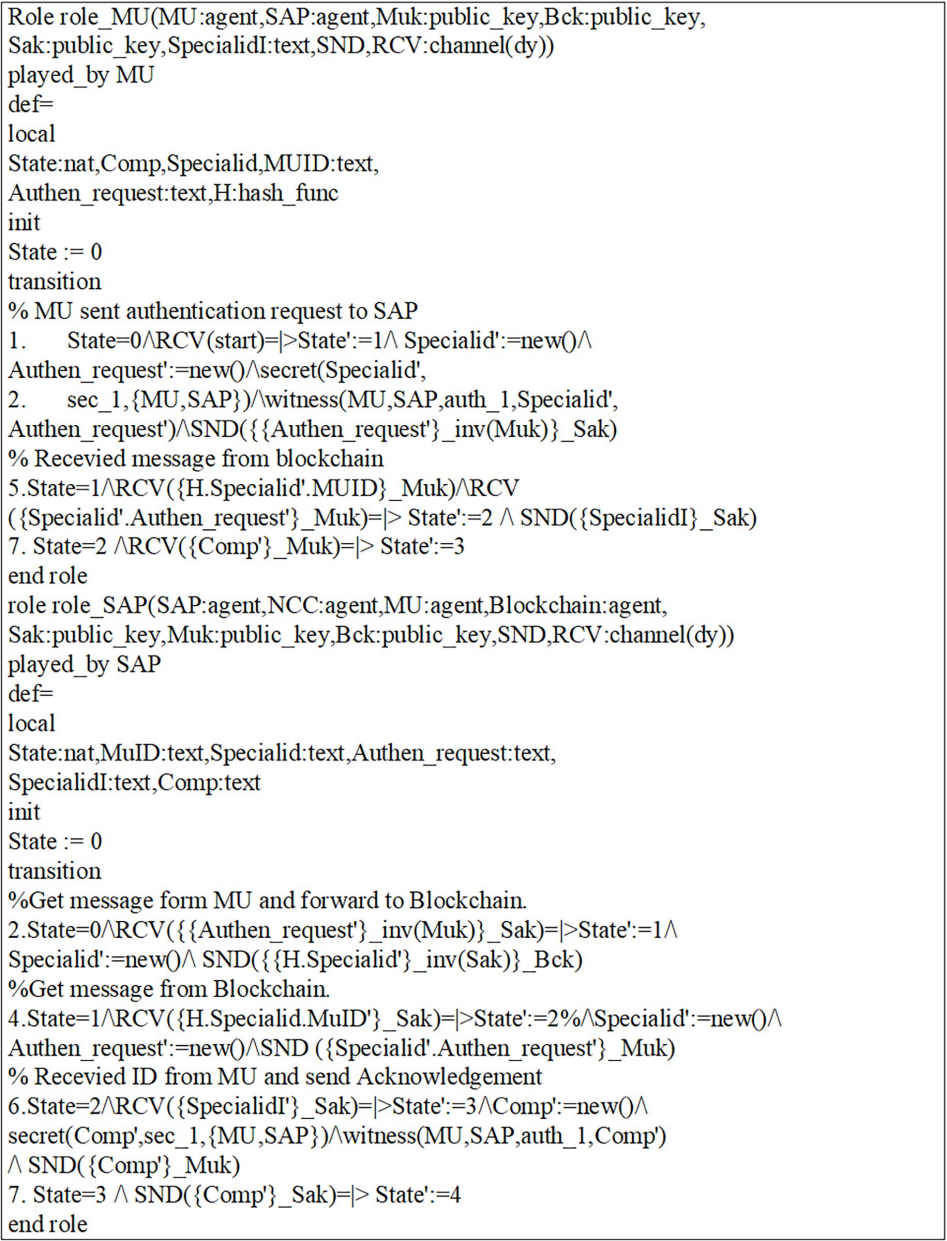
Role of MUs and SAP in HLPSL language.

**Fig 9:**
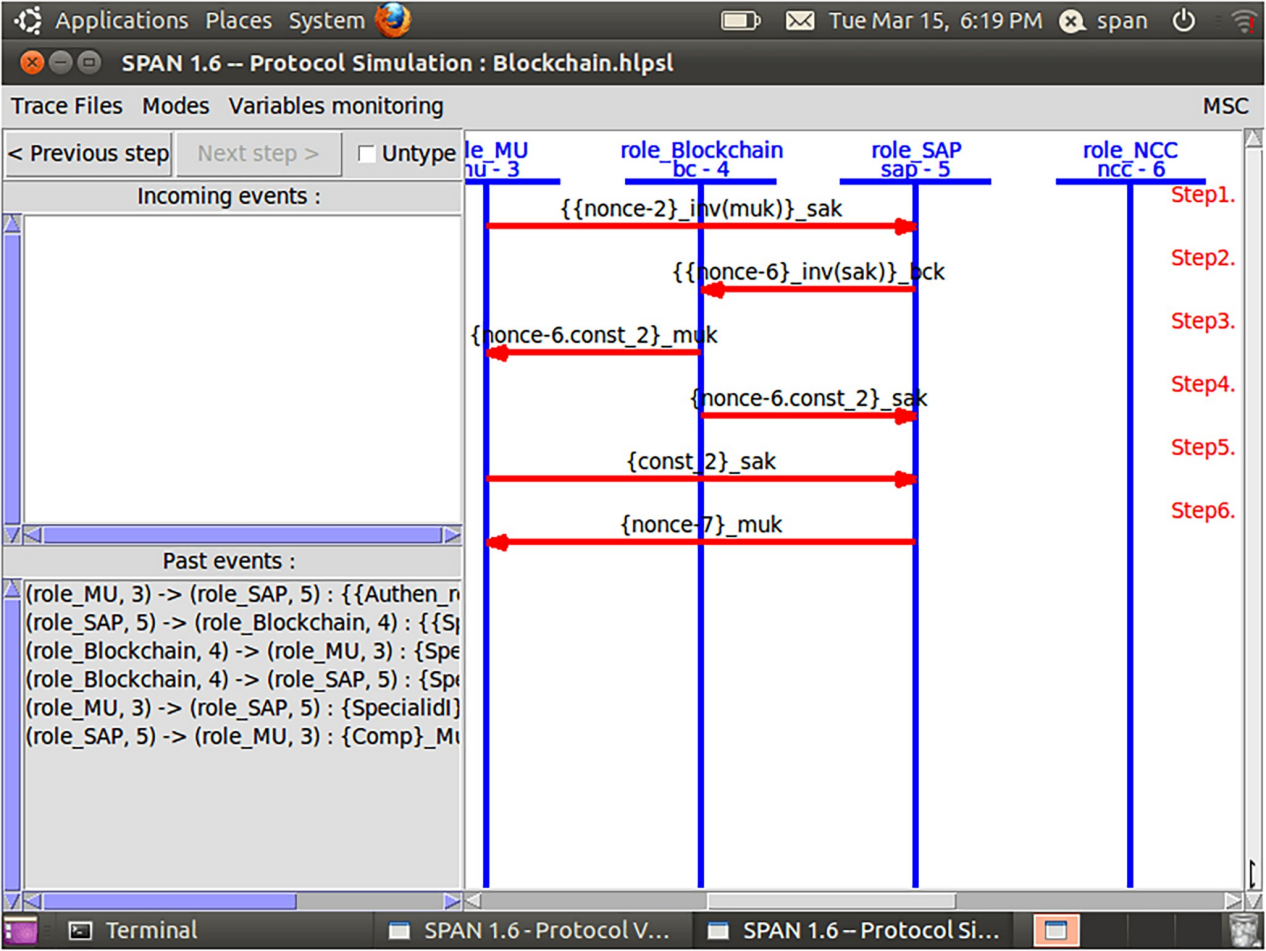
Verification diagram of blockchain-based mutual authentication between MUs and SAP using AVISPA tool.

**Fig 10:**
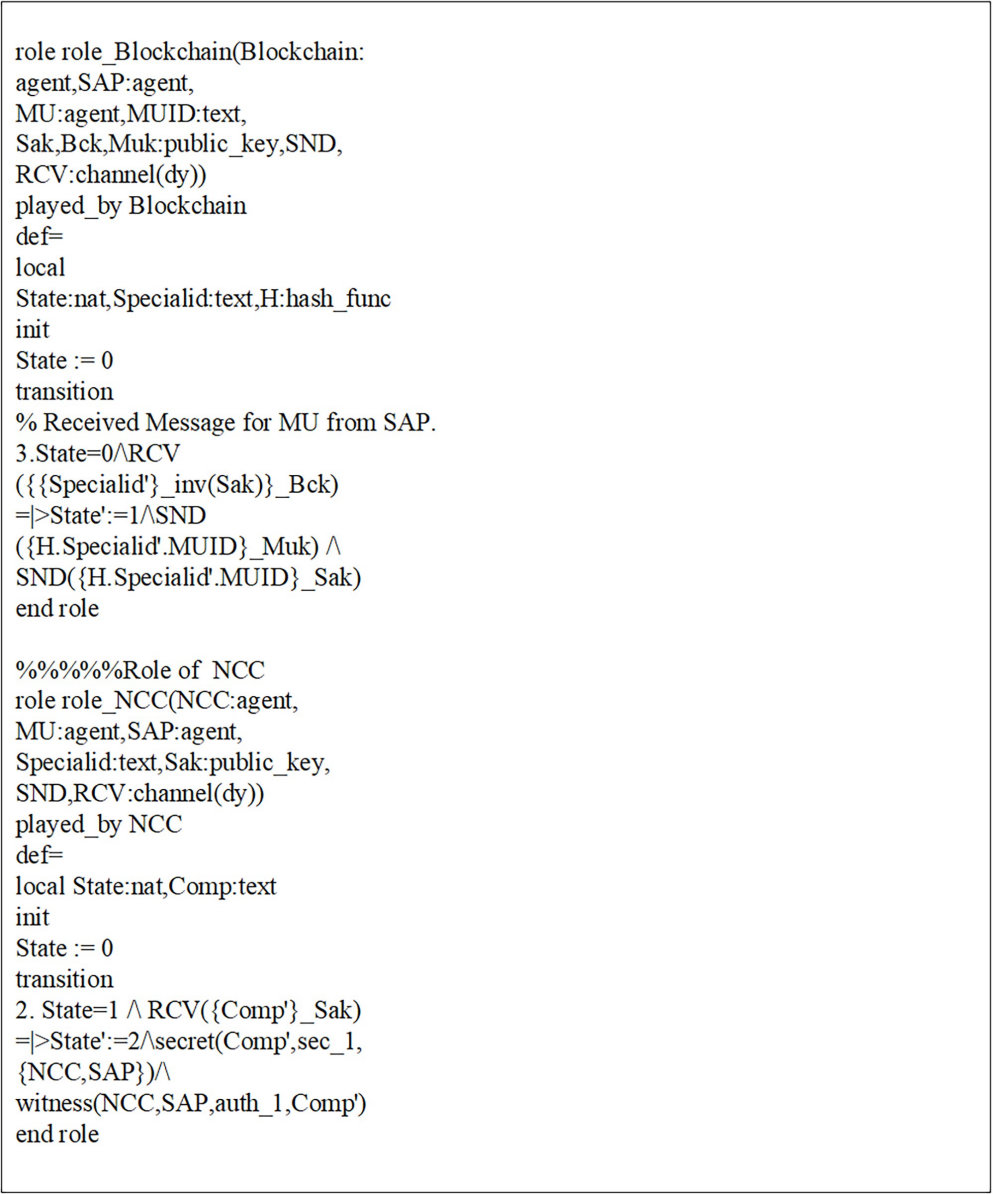
Role of blockchain and NCC in HLPSL language.

Initially, MUs and SAP registered their credentials with the NCC for blockchain-based mutual communication, as depicted in Figs 6 and 7. Now, the MUs are launching blockchain-based mutual authentication with SAP to gain access to SIN resources. Using the send() operation, the MUs transmit his/her authentication request to SAP over a secure channel. The transaction included an authentication request, a MUs private key, and a SAP public key. As shown in Figs 8 and 9., the SAP accepts the MUs authentication request and forwards the MUs authentication request to the blockchain to conduct a secure communication. Similarly, using the REC() operation, blockchain accepted SAP requests over a secure channel. As shown in Figs 9 and 10, the blockchain assessed the authentication request of (MUs, SAP) credentials before delivering verified credentials to SAP and MUs. The parameters include the hash function, another special identity for MUs, an SAP public key, and the MUs public key. In order to commence secure communication, MUs sent an acknowledgement message to SAP after acquiring a confirmation transaction from the blockchain. The acknowledgement message parameter is an SAP public key with a unique id. Furthermore, SAP has delivered a message stating that a blockchain-based mutual authentication procedure has been accomplished. The message carries the entire process (*Comp*) along with the MUs public key, as shown in Figs 9 and 10.

**Fig 11:**
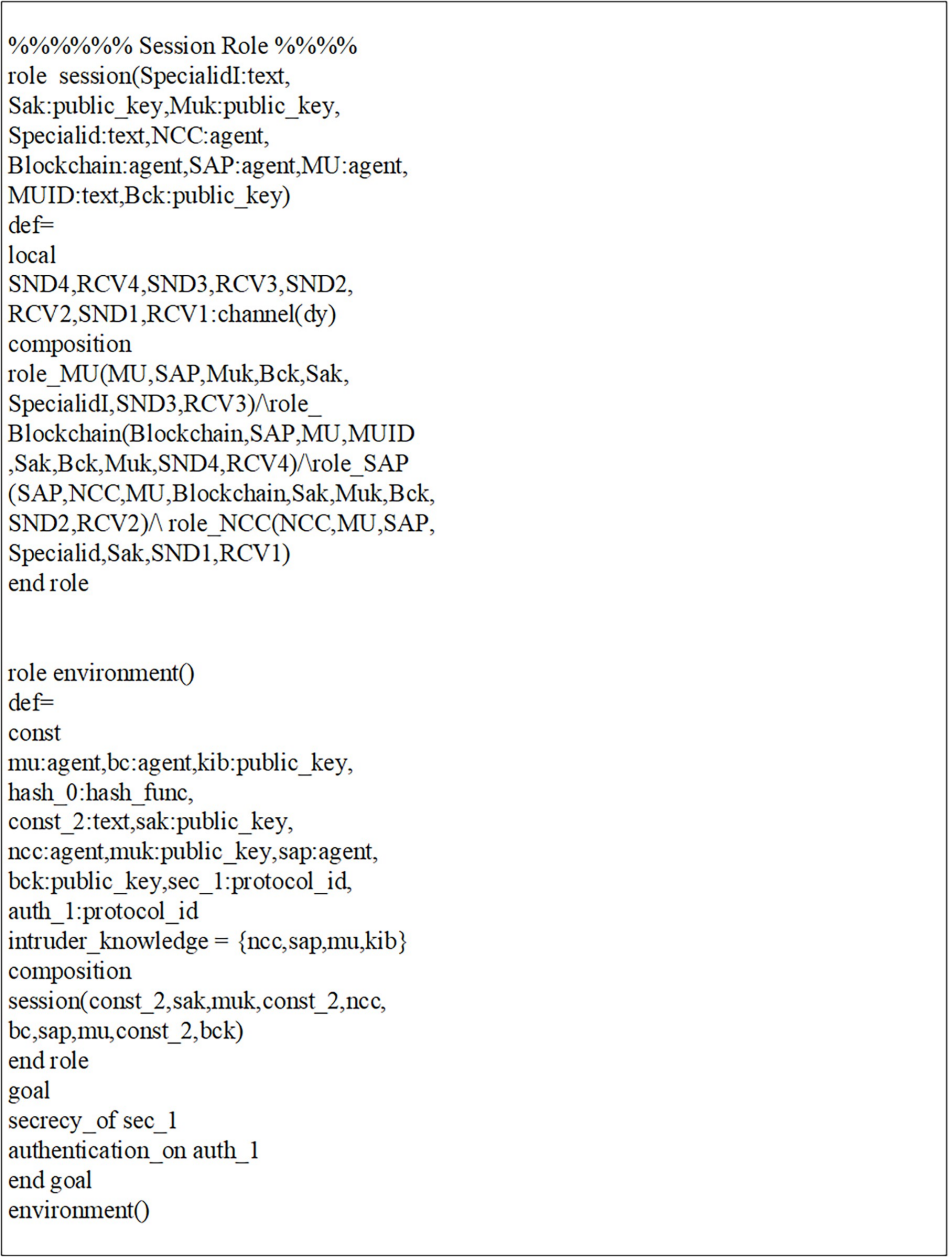
Role specification for the session, goal, and environment in HLPSL.

In addition, a session, goal roles, and environment, the roles of entities in HLPSL, namely, MUs, SAP, and blockchain Agents, are executed. In the specific role, the session role explains how to combine communicating entities. The environment role reveals that the recommended authentication protocol is peer-to-peer, as depicted in Fig 11. The goal’s role is to determine the security requirements for the mutual authentication technique that relies on blockchain.

**Fig 12:**
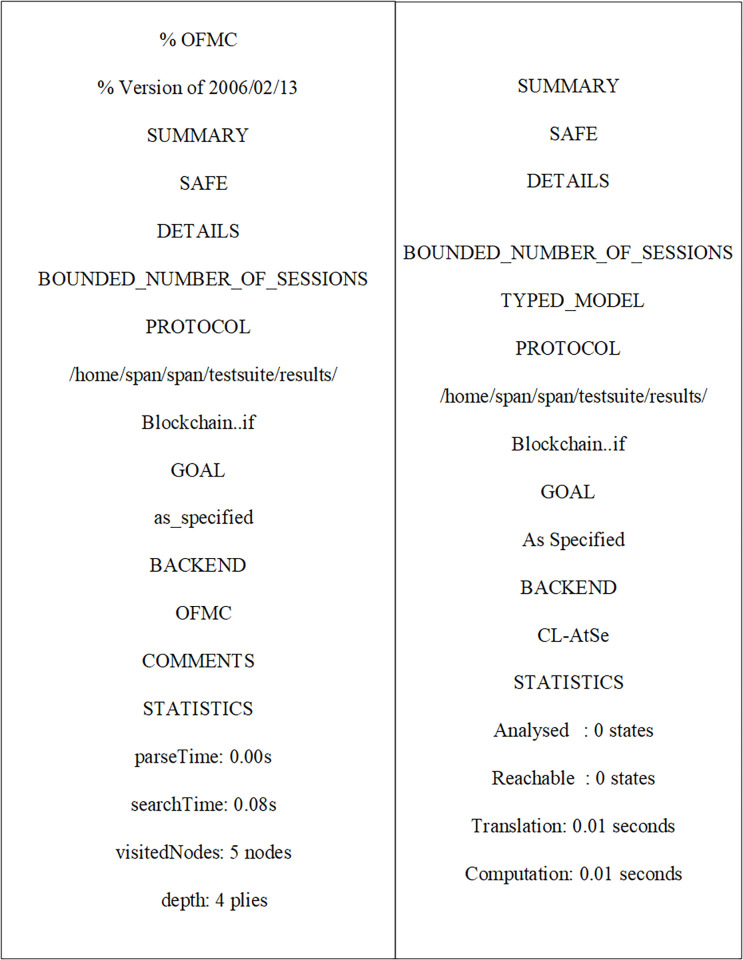
Verification results of blockchain-based mutual authentication between MUs and SAP using OFMC and CL-AtSe.

The mutual authentication algorithm is tested using the backends OFMC and ATSE (Attack Searcher). As shown in Fig 12, the results show that MUs with SAP authentication are safe and secure. Likewise, the registration phase demonstrates a secure registration of MUs and SAP, as demonstrated by Fig 6. As shown in Fig 12, five nodes are visited, and 4 are the depth of the search result. Furthermore, the cost of computation during algorithm verification is 0.01 seconds. This analysis provides encouraging assurance that the proposed blockchain-based mutual authentication protocol is safe and is compatible the design requirements for MUs in SIN. Eventually, the proposed algorithm is tested using the SPAN tool to identify and generate a Message Sequence Chart (MSC) that depicts possible attacks and adversary activities. Finally, verification results demonstrate that the proposed algorithm effectively defends against both replay and man-in-the-middle attacks.

## 8 Performance analysis

In terms of processors, power, and memory, MUs have limited resources. Thus, in wireless mobile contexts, efficient utilization of resources through communication and computing is a crucial challenge. The efficiency assessment is accomplished in particular in terms of communication complexities and computational cost.

The main objective of the protocol is to give users an authentication framework that is efficient, flexible, low-cost, and secure when combined with blockchain applications. To evaluate the performance of the blockchain-based authentication protocol using performance metrics. The following section compares the performance of a blockchain-based authentication algorithm to recent decentralized authentication schemes of SIN.

### 8.1 Verification

The verification feature in the proposed technique effectively checks the authenticity of MUs using a digital signature and a public key. To validate the public key, the MU digitally signs the message with the private key. The digital signature is validated using the MUs corresponding public key.

### 8.2 Security attacks

Blockchain technology is efficient at safeguarding the system from various security threats such as data integrity, authentication, and non-repudiation. In terms of MUs authentication, the blockchain is in place to verify MUs identities. The second feature of blockchain is immutability, which means that once data is stored in the server, it cannot be altered.

Besides that, transaction and function specifics are irreplaceable. Third, in SIN scenarios, our security protocol model always validates the authenticity of MUs. As a result, our scheme is effective against impersonation, replay, man-in-the-middle, denial-of-service attacks, and forward/backward secrecy, as shown in Table 4.

**Table 4:** Comparative study of security properties.

Security Prerequisites	Our Protocol	Kai et al’s [40]	Lee et al’s [49]	Liu et al’s [19]	Qing et al’s [17]
User Anonymity	√	√	√	√	√
Mutual authentication	√	√	√	√	√
Replay attack	√	√	√	√	√
Impersonation attack	√	√	√	√	×
Denial-of-service attack	√	×	√	×	×
Forward/backward secrecy	√	√	√	√	√
Man-in-the-middle attack	√	√	×	×	√

### 8.3 Signal overhead

The proposed algorithm signal overhead is determined by comparing the number of transmitted messages with the existing protocols [17, 19, 40, 49]. Table 5 demonstrates a comparison of signal overhead between recent authentication schemes. To fulfil mutual authentication in the proposed scheme, MUs and SAP require at least two exchange signalling messages, just like other protocols. Nonetheless, only one message must be exchanged between SAP and NCC, and no messages will exchange between a ground station and NCC. The proposed algorithm already received MUs acknowledgement from the blockchain for authentication.

**Table 5:** Comparison of signal overhead.

	SAP-NCC	SAP-G-NCC	MU-SAP
Lee et al’s [49]	4	4	2
Liu et al’s [19]	4	4	2
Kai et al’s [40]	1	1	2
Qing et al’s [17]	1	1	2
Our Protocol	1	0	2

As a consequence, the proposed scheme has a significant advantage over other authentication protocols. As shown in Table 5 all other authentication protocols must exchange at least two signaling messages between SAP and ground station, and two signaling messages between NCC and ground station. Since, the blockchain-based mutual authentication protocol outperforms existing protocols in terms of signaling overhead. Since, existing authentication schemes increase delay and overhead for MUs during SAP authentication, in contrast to the proposed protocol reduces burden on the ground station.

### 8.4 Authentication delay

The authentication latency is referred to as the total time consumed during the blockchain mutual authentication process, including computing and message propagation time. First, as shown in Table 6, we discuss the running time of various cryptographic operations. The following cryptographic symbols are used to compute computational overheads:

- *T*_*ho*_: A hash function’s running time.
- *T*_*enc*/*dec*_: Running time of the symmetric encryption and decryption function.
- *T*_*enc*/*dec*_: Running Time of the Asymmetric Encryption and Decryption Functions.
- *P*_*o*_: The number of point operations performed on ECC.

**Table 6:** Running time of different cryptographic operation.

Notation	Description	Running time(s)
*T*_*ho*_	(SHA-256) hash operation	0.0006
*T*_*enc*/*dec*_	Symmetric encryp/decryp	0.0088
*T*_*enc*/*dec*_	Asymmetric encrypt/decrypt	0.01825
*P*_*o*_	ECC point operation	0.763

As shown in the Table 6, three operations dominate the running time, asymmetric/symmetric encryption and decryption operations, hash operations, and number of point operations on ECC. Table 6. We follow the observation in [50] for an MNT curve to embedding degree 160 − *bit* − *q* and *k* = 6. The application was run on an Intel(R) Core(TM) i7-9750H processor running at 2.60GHz, yielding the following observation results:*T*_*ho*_: is 0.0006 ms, *T*_*enc*/*dec*_: is 0.01825 ms, *T*_*enc*/*dec*_: is 0.0088 ms, and *P*_*o*_ is 0.763 ms. The running time of message propagation between the MUs and SAP, SAP, and NCC has been denoted as *TMU* − *SAP*, *TSAP* − *G*, and *TG* − *NCC*, respectively. As the SAP altitude 499 to 1999 km from the ground station and NCC [49]. It is self-evident that *TSAP* − *G* = *TMU* − *SAP* = 10 ms. In order to compare authentication latency, we assume that the message propagation latency between a ground station and an NCC, *TG* − *NCC*, is 10 ms. SAP and MUs communicate directly in the proposed design, avoiding the need of a ground station.

Based on these assumptions, we compare the blockchain-based authentication protocol to the existing authentication protocols in terms of computation cost, transmission delay, and authentication latency from [19, 35, 40, 49] in Table 7. According to Table 7, the computational cost of the proposed protocol is higher than [19, 35, 49]. Since proposed algorithm employs costly elliptic curve cryptography operations. Nonetheless, the proposed algorithm has a lower computational cost (1.5994 ms) than [40]. Although the computational (cost) of the other algorithms are less than 0.001 ms.

**Table 7:** Comparative study of authentication delay.

	Transmission delay (ms)	Computational cost (ms)	Authentication delay(ms)
Lee et al’s [49]	4*T*_*MU*−*SAP*_ + 2*T*_*G*−*NCC*_(≡ 60)	10*T*_*ho*_(≡ 0.001)	60.001
Liu et al’s [19]	4*T*_*MU*−*SAP*_ + 2*T*_*G*−*NCC*_(≡ 60)	9*T*_*ho*_(≡ 0.0001)	60.0001
Chang et al’s [35]	4*T*_*MU*−*SAP*_ + 2*T*_*G*−*NCC*_(≡ 60)	10*T*_*ho*_(≡ 0.001)	60.001
Kai et al’s [40]	2*T*_*MU*−*SAP*_(≡ 20)	9*T*_*enc*/*dec*_ + 6*T*_*ho*_ + 4*P*_*o*_(≡ 5.4046)	25.5046
Proposed scheme	2*T*_*MU*−*SAP*_(≡ 20)	8*T*_*enc*/*dec*_ + 5*T*_*ho*_ + 2*P*_*o*_(≡ 1.5994)	21.5994

Moreover, All of these [19, 35, 49] authentication schemes have a longer propagation delay for MUs authentication in SIN, which is 60 ms, but [40] and proposed schemes share a similar (20 ms) transmission delay. In general, NCC is responsible for MUs verification in [19, 35, 40, 49], so that MUs confidential information (e.g., real identity) is not leaked to any foreign network entities. In in order to compare authentication latency, we compute propagation latency among MUs, NCC, and SAP.

Due to less signaling message transmission between MUs and SAP/NCC, the proposed algorithm have reduced propagation latency for overall authentication compared to current algorithms. As a result, the authentication delay has been reduced significantly. Table 7, shows that the overall authentication delay of other schemes [19, 35, 49] is 60.001ms, while the [40] scheme is only 25.5046ms. However, the proposed authentication has a lower authentication delay (21.59994 ms) than [40], which has a delay of 25.5046 ms. Finally, the proposed blockchain-based authentication algorithm has been more effective in offering MUs with robust and efficient access authentication for SIN.

### 8.5 Running time

In terms of SIN running time, the proposed blockchain-based authentication protocol outperformed as compared to existing authentication protocols. The outcome shows that our proposed blockchain-based scheme takes less time running than [17, 40]. The results show that blockchain-based MUs authentication takes less time than the current protocol. It is also worth noting that as the number of MUs increases, our scheme performs better in terms of time complexity. If MUs want to access SIN, they must first sign a message with a digital signature known as a private key. SAP verifies the public key against the digital signature after receiving the signature. Furthermore, our proposed scheme makes use of MUs public key, which is verified only once in the NCC. As a result, the proposed scheme has a shorter running time than current SIN authentication protocols.

### 8.6 Assessment of group authentication

**Fig 13:**
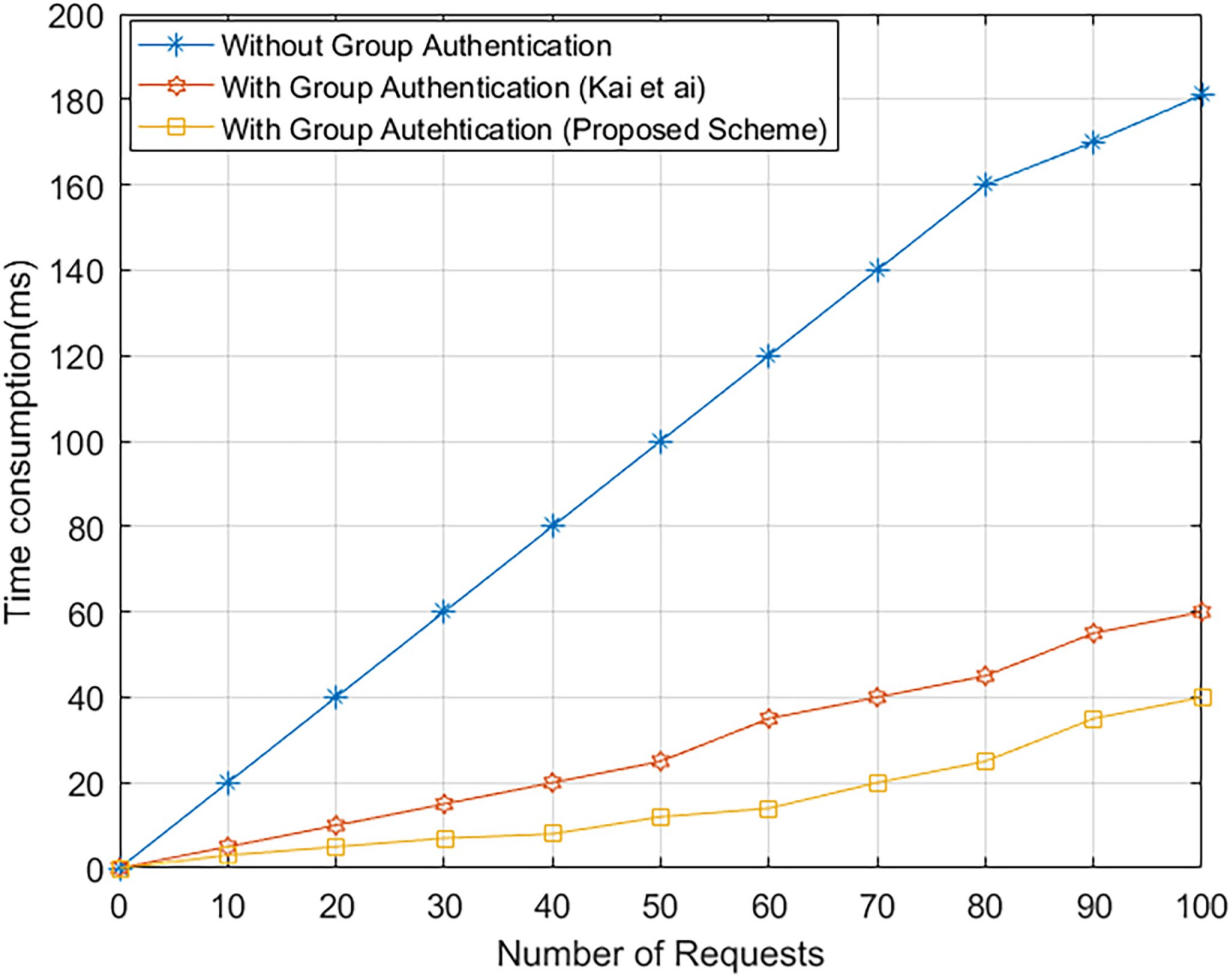
With/without group authentication computational cost.

Whenever, a SAP accepts a large number of authentication requests from multiple MUs, group authentication can significantly reduce the SAP/computational LEO’s cost. Table 8, shows the computational overhead of one authentication, n authentication except for the group signature, and n authentication with the group signature. As shown in Fig 13, the line graph describes the number of authentication requests computational overhead for with/except Group signature. The coordinate specifies the respective computational overhead with/without authentication, ranging from 0 to 100. The proposed blockchain-based group signature has a significantly lower computation overhead than Kai et al’s [40] Group signature authentication algorithm, as demonstrated in Fig 13. When N MUs forward their authentication requests concurrently, the computational cost can be drastically reduced by using group authentication.

**Table 8:** Comparative study of cost verification.

	Computational Cost
One Request	3*T*_*enc*/*dec*_ + 2*T*_*ho*_ + 2*P*_*o*_
n authentication request without Group verification	3*nT*_*enc*/*dec*_ + 2*nT*_*ho*_ + 2*nP*_*o*_
n authentication request with Group verification	(*n* + 2)*T*_*enc*/*dec*_ + 2*nT*_*ho*_ + 2*nP*_*o*_

## 9 Conclusion

In this article, we presented the access authentication scheme as a security essential of SIN and uses of blockchain applications using a well-organized technique. To improve the quality of communication in SIN, the proposed blockchain-based scheme offers major security attributes and lower delay for mobile users. Meanwhile, the integration of blockchain into the algorithm enhances access authentication when there is a significant number of mobile users. The simulation results and security investigation demonstrate that our algorithm is more secure in confronting of different attacks such as man-in-the-middle and replay attacks. Finally, the proposed authentication algorithm is simulated and verified utilizing the AVISPA tool, and the performance study illustrates that the new algorithm is more efficient than current authentication algorithms in terms of overhead, authentication latency, time complexity, and group authentication computation cost. Consequently, it is appropriate for a secure space communication environment system. In future, as the number of nodes in SIN grows, researchers will be required to minimize communications overhead.
